# Recent Advances in Supercritical Fluid Extraction of Natural Bioactive Compounds from Natural Plant Materials

**DOI:** 10.3390/molecules25173847

**Published:** 2020-08-24

**Authors:** Pascaline Aimee Uwineza, Agnieszka Waśkiewicz

**Affiliations:** Department of Chemistry, Poznan University of Life Sciences, Wojska Polskiego 75, 60-625 Poznań, Poland; agat@up.poznan.pl

**Keywords:** bioactive compounds, supercritical extraction, supercritical fluids, co-solvent, essential oils, medicinal plants

## Abstract

In this review, recent advances in greener technology for extracting natural bioactive components from plant origin sources are discussed. Bioactive compounds of plant origin have been defined as natural chemical compounds present in small amounts in plants. Researchers have shown interest in extracting bioactive compounds because of their human health benefits and characteristics of being eco-friendly and generally recognized as safe. Various new extraction methods and conventional extraction methods have been developed, however, until now, no unique approach has been presented as a benchmark for extracting natural bioactive compounds from plants. The selectivity and productivity of traditional and modern extraction techniques generally depend on selecting the critical input parameters, knowing the nature of plant-based samples, the structure of bioactive compounds, and good scientific skills. This work aims to discuss the recent advances in supercritical fluid extraction techniques, especially supercritical carbon dioxide, along with the fundamental principles for extracting bioactive compounds from natural plant materials such as herbs, spices, aromatic and medicinal plants.

## 1. Introduction

Extraction is one of the approaches used to isolate components from plant-based materials. At present, various extraction methods are used at the laboratory, pilot, and commercial scales by numerous researchers to extract the different target compounds present in plants [1]. Bioactive compounds are natural secondary metabolites extracted from various plant parts, such as leaves, stem, roots, seeds, flowers, and fruits, by using several extraction procedures [2,3]. Demand for these compounds has increased because they are perceived as natural and safe for applications in numerous industries such as cosmetics, food, feed, agriculture, and pharmaceuticals [3,4]. Bioactive compounds have been found to possess a wide spectrum of health-promoting properties for humans and animals such as antibacterial, antimicrobial, anti-inflammation, anti-aging, and anti-cancer effects [2,4,5,6].

Bioactive compounds—essential oils, carotenoids, fatty acids, phenolic acids, flavonoids—were conventionally extracted by steam distillation, solvent extraction, Soxhlet extraction, pressing method, and hydro-distillation, but they exhibit some limitations such as being too time-consuming, using too much organic solvents, losing some volatile compounds, degrading thermolabile compounds, the possibility of leaving toxic solvent residues in the extract, low yield, and low extraction efficiency [3]. Therefore, in recent years, green chemistry methods were developed for extraction purposes to reduce energy and solvent consumption, reduce processing times, and replace conventional solvents with eco-friendly substitutes.

Modern approaches to extract and isolate bioactive compounds from plants-based materials are gaining attention in the research and development fields. For example, ultrasound-assisted extraction (UAE), pressurized liquid extraction (PLE), supercritical fluid extraction (SFE), and microwave-assisted extraction (MAE) [7,8,9,10] are currently accessible and environmentally sustainable technologies. According to Chemat et al. [11], greener technology is defined as “extraction methods based on the detection and development of extraction processes which will reduce energy consumption, enables the use of solvents substitutes, renewable natural products, and ensure a safe and high-quality extract/product” [11].

Ultrasound-assisted extraction is a mechanical technique based on sound waves, frequencies, and amplitudes that stimulate cell wall disruption and the discharge of cell content [8,10,12]. This process reduces the extraction time, is suitable for thermolabile and unstable compounds, improves the extraction yield, and reduces the solvent and energy consumption because of its effect on the transport mass, temperature, and solvent/sample ratio [8,10]. Pressurized liquid extraction is a solid–liquid technique consisting of the application of high pressure and temperature that raises the solvent’s boiling point and induces its quick penetration into the sample matrix [7]. PLE contributes to a lower use of solvent, shorter extraction time, and increased extraction yield. However, it is not recommended for heat-sensitive compounds due to the high extraction temperature [7,10]. Supercritical fluid extraction is an advanced technique for extracting bioactive compounds by employing supercritical fluids as solvent. It has gained much attention over traditional methods due to its significant benefits, such as higher selectivity, diffusivity, and ecology [13].

Among the novel technologies mentioned, we discuss in this review supercritical fluid extraction (SFE) which is one of the best techniques for removing natural chemical components such as flavonoids, essential oils, seed oils, carotenoids, and fatty acids from natural plant materials, and it represents a sustainable alternative to traditional extraction systems [10,13]. Researchers have shown the considerable benefits of SFE over conventional techniques [3,9,10]. Carbon dioxide (CO_2_) is an excellent solvent that has received particular attention in SFE because it is chemically inactive, economical, easily accessible, separable from extracts, non-toxic, and is an approved food-grade solvent [7,9]. Supercritical carbon dioxide (SC-CO_2_) is a nonpolar solvent that is frequently used in SFE due to its gas-like and liquid-like properties, low critical temperature and pressure, and it has the selectivity and potentiality to extract heat-sensitive compounds. Furthermore, low polarity compounds and small molecules are easily dissolved in SC-CO_2_, but large molecules and polar compounds are extracted with the addition of a co-solvent to enhance the extraction yield, which can be ethanol, methanol, or water. A particular focus should be placed on temperature and pressure during SFE because any alteration affects the entire process [3,13].

The objective of this paper was to provide an upgraded review on the claims of SFE by discussing the characteristics and principles of SC-CO_2_, its applications in extracting natural bioactive compounds from plant-based materials, and its current use in different industries.

## 2. Characteristics of Supercritical Fluids as a Novel Extraction Technique

### 2.1. Background of Supercritical Fluid Extraction 

SFE is classified among the novel extraction techniques that are a more environmentally friendly method by which to produce indigenous substances that have applications in various industries from sustainable sources such as herbs, spices, aromatic and medicinal plants. This advanced technology consists of the isolation/removal of targeted bioactive compounds through supercritical fluids.

In 1822, the initial discovery of the supercritical phase was realized by Baron Charles Cagniard de la Tour, who noticed changes in solvent behavior at a particular value of pressure and temperature [14,15]. The term "critical point" was coined by Thomas Andrews in 1869 as a result of his experiments on the effects of temperature and pressure on as sealed glass tube of partly liquefied carbonic acid. He described it as the endpoint of the phase equilibrium curve which the critical temperature (Tc) and critical pressure (Pc) reached when the existence of two phases disappeared [10,14]. A few years later, Hannay and Hogarth discovered the SFE method application and the fundamentals of this technology using CO_2_ in the supercritical state were developed in 1960 [16]. The earliest practical application of supercritical fluids was the decaffeination of green coffee beans started in Germany; after a few years, the extraction of oils from hops using liquid CO_2_ was developed in Australia [17]. By the 1980s, industrial applications of both technologies were developed and effectively adopted in different countries [16]. Currently, various products are being produced using the technology and are accepted all over the world.

### 2.2. Concept of Supercritical Fluids Extraction and Principles

Basically, a simple SFE process comprises extraction and separation as the essential steps [17]. During extraction, either solid or liquid samples may be used, depending to the system settings, but solid samples are more used compared to liquid samples. Regarding solid samples, columns are filled with pre-treated (dried and milled) samples, and the pressurized supercritical solvents flow through the column and dissolve extractable compounds from the solid matrix. The dissolved compounds are transported by diffusion out to the separator where the mixtures of extract and solvent are separated through pressure reduction, temperature increase, or both [17,18,19].

Many companies are now commercially producing SFE equipment at varying scales according to their intended use, such as for use in the laboratory, pilot studies or industrial use. The most simple system consists mainly of a chiller used to cool the solvent gas, a solvent pump that pushes the fluid throughout the system, an extraction column that holds the samples to be extracted, separators which collect the extract, heat exchangers for adjusting the temperature of process materials, an oven utilized to keep the extraction column above the critical temperature of the extraction fluid, and a back pressure regulator used to maintain the pressure in the system above the critical pressure of the fluid [20,21]. SFE can be implemented in two different modes—dynamic and static—which can be used separately or combined during extraction. In dynamic mode, the supercritical fluid flows steadily through the extraction column containing the sample, while in static mode, the sample absorbs the supercritical fluid and there is no run-off fluid from the extraction column during the process [22].

Most of the time, co-extraction of unwanted compounds occurs during the extraction process, which might result in a poor-quality extract. Thanks to advanced technology, this problem can be solved by conducting SFE with a fractional separation process, allowing the improvement of selectivity in SFE [23]. This concept can be conducted in multiple steps during separation, by coupling some separators in series and adjusting processing conditions, such as pressure and temperature, according to the equilibrium solubilities of the targeted compounds [24].

During SFE, different variables, such as extraction temperature, pressure, type, percentage of co-solvent, and the sample size must be optimized to enhance the extraction yield of targeted compounds. Additionally, the solubility and mass transfer resistance of raw materials are associated with those variables [25,26,27]. The solubility of an extractable compound must be maximized in SFE because it is one of the main factors influencing the effectiveness of extraction and the quality of extract [13]. It has been reported that the solubility of the solute in SFE depends on temperature and pressure, which have an effect on the density of fluid. SFE is a convenient technique because it enables an adjustment of the solvent power or selectivity of the supercritical fluids (SCF), and it can be directly connected with gas chromatography (GC) or supercritical fluid chromatography for analytical purposes [27]. Various studies have shown numerous benefits of SFE including its fast processing time, suitability for extracting volatile and thermolabile compounds, higher productivity in terms of increased yields, reduced solvent use, and protection of the environment by using safe solvents. It plays a vital role in different areas such as food, pharmaceutical, agriculture and cosmetics [18]. As discussed, nowadays SFE is not only applied in laboratories for research purposes but its application has been developed commercially at an industrial scale, for example, to produce natural food ingredients (hops, aromas, spices, colorants, vitamin-rich extracts, specific lipids), nutraceuticals, pharmaceuticals, and to remove pesticides from food products [27,28]. The use of supercritical fluid is characterized by different properties, namely, density, viscosity, and diffusivity, which can be changed to improve its transport properties.

### 2.3. Properties of Supercritical Fluids

Supercritical fluids are chemical solvents that can be compressed above their critical point, are generally considered environmentally friendly, and are commonly used in the extraction process because they provide excellent results due to their unique characteristics [29]. SCF is used as a replacement for organic solvent in laboratory processes and various industries such as food, pharmaceuticals, agriculture, and cosmetics. As shown in Table 1, many compounds have been considered as SCFs, for example, hydrocarbons (pentane, butane, hexane), aromatics (benzene, toluene), alcohols (methanol, isopropanol, *n*-butyl alcohol) and some gases (carbon dioxide, ethylene, propane) [18]. Among the abovementioned SCF, CO_2_ is unquestionably the most often employed solvent due to its numerous different benefits [25,29].

A fluid is regarded as supercritical when its pressure and temperature are beyond its critical points, that is, critical pressure (Tc) and critical temperature (Pc). In this state, the fluid is represented by both its gas and liquid phase properties in an advantageous way. Their actions are close to that of gas in some ways, but close to liquid in others [30].

**Figure 1:**
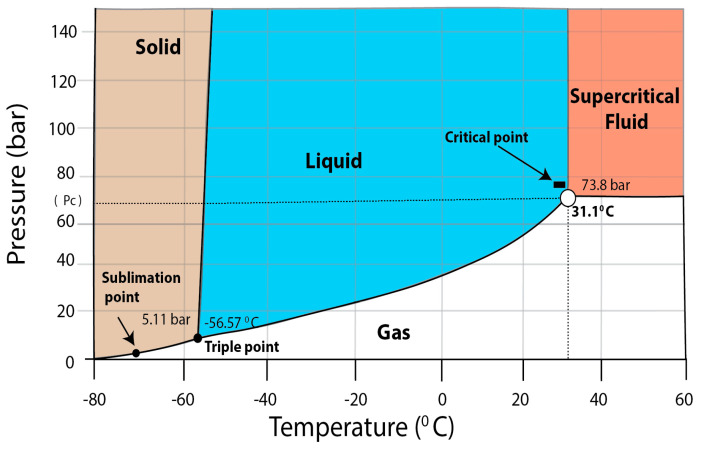
Scheme p-T representation of the CO_2_ phase based on Gopaliya et al. [32] with own modifications.

For example, a simple CO_2_ phase diagram can demonstrate the concept of the supercritical state (Figure 1). CO_2_ usually exists in three physical states (solid, liquid, and gas) due to variations of pressure and temperature [29], but sometimes there is a fourth CO_2_ phase—supercritical fluid—that is the result of the dynamic equilibrium that appears at the point where there are no differences between liquid and gas phases [31,32]. The phase diagram below shows different points and states of CO_2_ in terms of combinations of high or low temperature and pressure. The triple point on this diagram is the point that represents all three phases of CO_2_ together in equilibrium (−56.6 °C and 51.1 bar) [32]. The critical point appeared at 31.1 °C and 78.3 bar, which are the Tc and Pc of CO_2_, respectively [25,29,30]. The region above the critical point is a supercritical fluid region which appears when there is no physical difference between a gas and a liquid, and over that critical point no matter how much pressure or temperature is increased, the substance cannot be changed from liquid to gas or from gas to liquid [30,32]. 

Briefly, the supercritical region can be attained through two approaches: (i) by increasing the pressure above the Pc-value of the substance, while maintaining a stable temperature and then extending the temperature above the Tc-value at a stable pressure value; or (ii) by increasing the temperature above the Tc-value first and then increasing the pressure above the Pc-value. 

Supercritical fluids generally display physicochemical properties between those of a liquid and gas, typically characterized by low viscosity, high density, and diffusivity, which can be managed by changing the pressure and/or temperature (Table 2) [29,33,34]. Those properties are predominant during the SFE process design and make them an excellent solvent for different applications [17] due to their penetrative power, which is based on the high mass transfer rate and high density of fluids in the extractable components.

The density of a supercritical fluid is between that of a gas and a liquid but is closer to that of a liquid. It mostly relies on pressure and temperature conditions: When pressure rises at a constant temperature, it increases the density of a supercritical fluid and solvating power. On the other hand, when the temperature rises at a constant pressure, it reduces the density of a supercritical fluid and solvent strength [32]. Therefore, this is the main characteristic and fundamental source of the excellent dissolving properties of fluid with a solute molecule, which is very strong.

The viscosity of a supercritical fluid is one of the thermophysical properties of SCF which is generally known to be low and nearly equal to that of a gas but less than that of a liquid [17]. This low viscosity enhances the penetration power of SCF due to the lower resistance than that of a liquid to a solute. Temperature has a higher impact on SCF viscosity than liquids [32].

The diffusivity of a supercritical fluid is typically higher than in a liquid and lower than in a gas [17]; consequently, the solute can show better diffusivity in a SCF than in a liquid. Moreover, temperature and pressure conditions show different effects: as the pressure increases, the diffusivity in a SCF decreases and when the temperature increases, the diffusivity also increases. This property enhances the capacity of SCF to be a suitable solvent for analytical purposes [32,33].

As mentioned previously, these properties are connected, and make SFE stand out as an excellent technique. For example, low viscosity and high diffusivity allow better transport properties than liquids; they diffuse effortlessly in a solid sample and increase efficiency and extraction yield. Additionally, high density combined with solvent power can be tailored automatically, and this can give high solubility and high selectivity to the SCF; thus, new compounds can be extracted and employed in the development of new products [17].

## 3. Operational Use of Carbon Dioxide in Bioactive Compound Extraction 

Carbon dioxide is extensively used as the supercritical fluid in various fields for extraction purposes due to its critical properties (Tc = 31.1 °C; Pc = 73.8 bar), which makes it an excellent solvent for extracting bioactive compounds sensitive to heat [29]. The unique solvent properties of supercritical carbon dioxide have made it a desirable compound for separating antioxidants, pigments, flavors, fragrances, fatty acids, and essential oils from plant and animal materials. SC-CO_2_ has numerous benefits. It is a cheap and readily available in large quantities with a high degree of purity [28], furthermore it is an environmentally friendly substitute for organic solvents and is designated as a safe solvent by different organizations such as the U.S. Food and Drug Administration (FDA) and the European Food Safety Authority (EFSA) [35,36].

A further advantage of SC-CO_2_ is its ability to produce extracts free of solvent residue because CO_2_ is a gas at ambient temperature and pressure, so it can use a low amount of solvent and minimize thermal damage to bioactive compounds due to its low critical temperature.

CO_2_ has exciting features as a solvent because it has high diffusivity, it is safe for human health and the environment, reusable, inert, non-toxic, non-flammable, and non-corrosive [25]. CO_2_ is useful in supercritical fluid extraction processes because being close to the ambient environmental conditions it can readily penetrate through plant material and dissolve the targeted extracts. However, its extractability with substances depends on the occurrence of specific groups in the structure, as well as their polarity and molecular weight [13,35,37]. Additionally, the extract composition for volatiles or non-volatiles can be changed easily by modifying the pressure and temperature conditions. 

CO_2_ is gaseous at ambient pressure and temperature, which makes analyte recovery quite simple and provides solvent-free analytes. However, it has a limited solubility towards compounds because it is regarded as non-polar [38]. Studies have shown that hydrocarbons and other organic compounds with relatively low polarity and molecular weights (MWs) under 250 exhibit excellent solubility in SC-CO_2_. For example, esters, aldehydes, ethers, ketones, lactones, and epoxides are easily extractable in SC-CO_2_, so the process can be carried out at lower pressures (75–100 bar). Moderately polar components with an MW in the range of 250–400, such as benzene derivatives, substituted terpenes, sesquiterpenes, or oleic acid, are moderately soluble in SC-CO_2_, and a higher pressure is required for their extraction. Highly polar substances (MW over 400) that contain carboxylic acids and three or more hydroxyl groups (e.g., sugars, tannins, proteins, waxes, carotenoids, or pesticides) are almost insoluble in SC-CO_2_ [13,17]. To obtain the targeted compounds, a polar co-solvent such as methanol, ethanol, and water is generally used to enhance the dissolvability of polar compounds in the supercritical mixture and improve the selectivity of SC-CO_2_ [25,38].

## 4. Parameters to Consider and Their Effect during Supercritical Fluid Extraction 

During SFE, the choice of working conditions depends on the target compounds to be extracted; however, the parameters of temperature, pressure, co-solvent concentration, extraction time, and particle size are the most prominent and should be taken into consideration for efficient extraction.

### 4.1. Effects of Temperature and Pressure 

The extraction requirements of temperature and pressure are the primary parameters that influence the extraction efficiency because of their effect on the solubility of a substance during SFE. Generally, an increase of pressure at a specific temperature in the process increases the density of the solvent and the solubility of targeted compounds [28,39]. Therefore, the higher the pressure, the smaller the solvent volume needed for a particular extraction [28]. Nevertheless, elevating pressure to a given point can reduce the extract’s antioxidant activity and decrease the diffusivity of the SCF, which reduces solute dissolution [40]. Furthermore, a high pressure is not suggested for all substances and targeted compounds because it can result in a compacted raw material, which can adversely affect the extraction yield [41]. The extraction temperature at constant pressure has two opposing effects during SFE. Increased temperatures decrease the solvent density, thus reducing its solvating power, but it improves the vapor pressure of desired compounds, consequently increasing the analyte solubility and extraction yield. However, because increased temperature decreases the solvent density, this reduces its solvating power and analyte solubility, negatively affecting yield [28,40,42]. These two opposite effects may result in the cross over of the isotherms, in a phenomenon known as retrogradation, where the solubility of solute does not depend on the density of solvent [43]. Therefore, according to the literature, the supercritical fluid extraction temperature of thermolabile compounds has to be fixed between 35 and 60 °C to avoid degradation [27], and the pressure should be around 400 bar. These conditions should be carefully selected based on the purpose of the process and the nature of the targeted compounds [15].

Studies on the influence of temperature and pressure on the extraction process of bioactive substances gave different results due to the plant materials and targeted compounds [40,42,44,45,46,47,48]. For example, high pressures were reported to enhance the global extraction yield of phenolic compounds [40,45,47], while Akay et al. [46] reported a high total phenolic content under low pressure and no significant difference to the yield when increasing temperature and pressure. Some results showed that an augmentation in the recovery of phenolic compounds could be obtained by reducing the extraction temperature and increasing the addition of co-solvent and pressure [45]. 

### 4.2. Effects of Co-Solvent/Modifier

By definition, a co-solvent is an organic solvent that, when added at various proportion to CO_2_, may dissolve with the supercritical fluid, and can retain a considerable solvent power towards the targeted compounds [49]. As previously reported, CO_2_ is the most frequently used supercritical fluid in various industries, but its low polarity restricts its use for the extraction of lipophilic and polar compounds [15]. To overcome this problem, polar co-solvents such as ethanol, methanol, water, acetic acid, formic acid, and many other polar or nonpolar co-solvents, can be used during the extraction to improve the solvation power of SC-CO_2_, enhance its affinity for poorly soluble solutes (alkaloids, phenolics, and glycosidic compounds), increase solubility, and increase the extraction yield [25]. Among the listed co-solvents, methanol and ethanol are frequently used at concentrations below 10% of the quantity of CO_2_ employed for the extraction [15,37]. However, ethanol is less toxic than methanol. The best co-solvent for a specific extraction process should be determined through preliminary experiments, and the type of sample, targeted compounds, and its final application should also be considered.

Different researchers have shown that the co-solvent can affect not only the total extraction yield but also the bioactive properties of the extracts such as total phenolics extraction yield, antioxidant and anti-inflammatory activities of extracts [50,51,52]. The percentage and type of co-solvent are significant in the extraction. Both are crucial factors affecting the solubility of targeted compounds in SFE. However, the addition of a large volume of co-solvents can modify the critical parameters of the solution and can reduce the selectivity [51]. In SFE, there are three different ways that co-solvents can be used in the system: as a mixed fluid in the pumping system; by injecting the co-solvent as a liquid into samples before extraction; and as a cylinder tank of pre-modified CO_2_, although this method is expensive and seldom used [41].

Several scientists have studied the effect of co-solvent on SC-CO_2_ extraction, and their results have various implications. For instance, in terms of the carotenoid composition of pumpkin in SC-CO_2_ extraction with and without co-solvent, co-solvent increased the total carotenoids yield from pumpkin. Shi et al. [47] demonstrated that by increasing the polarity of CO_2_ with ethanol, it is possible to increase the total carotenoid yield by 1.8 times. In other studies, Uquiche et al. [51] investigated the impacts of ethanol concentration and pressure on the phenolics extraction yield, total extract yield, antioxidant and anti-inflammatory activities by using supercritical extraction of *L. rivularis* stalks. The result showed that the highest total extract yield, phenolics extraction yield, and antioxidant activity were achieved with low use of co-solvent, while anti-inflammatory activity improved with a greater addition of ethanol. Klein et al. [53] evaluated the effects of the addition of two different co-solvents (ethyl acetate and ethanol) and the operational pressure on the SC-CO_2_ extraction process, and the results revealed that the addition of ethanol as a co-solvent resulted in higher yields than those obtained using ethyl acetate.

### 4.3. Effect of the Raw Matrix on Supercritical Fluid Extraction 

Several elements, such as the nature of the raw material, moisture content, particle size, shape, surface area, and porosity are crucial elements that influence solubility and the mass transfer process during SC-CO_2_ extraction [13]. The correct selection of these elements can produce the complete extraction of the targeted compounds in a shorter time [27].

Typically, the sample material to be extracted must be dried to reduce the moisture content, as the water content in the sample can compete with the extractable solute to associate with the solvent and reduce the extraction yield; nevertheless, in some cases the presence of water is needed to permit the good interaction of solvent with the solute. The recommended moisture content should be between 4–14% [18]. The particle size of solid samples and porosity have a direct impact on the mass transfer rate in the process. Reducing the particle size increases extraction efficiency because diminishing particle size reduces the diffusion path of the solvent and increases the contact surface area which resulted in the acceleration of extraction process [15,18,41]. However, it is necessary to avoid overly small particles because they can increase the internal mass transfer resistance, which provokes channeling within the column and a reduction of efficiency and yield during the extraction process [15,18,27]. According to the literature, the mean particle size for SFE of natural products varies from 0.25 to 2.0 mm [15,18]. Different researchers studied the effect of particle size during SC-CO_2_, and Del Valle and Uquiche [54] reported that the oil yield was dependent on the particle size of the rosehip seeds. Aris et al. [55] investigated the effect of particle size on *Momordica charantia* extract yield with different mean particle sizes of 0.2, 0.3, 0.5 and 0.7 mm under constant operating conditions (200 bar, 65 °C and 4 mL/min), and showed that a reduction in the particle size could increase the yield. They concluded by showing that 0.3 mm was the best mean particle size to produce the highest *Momordica charantia* extract yield. Kehili et al. [56] investigated the influence of particle size of tomato peel on the oleoresin extraction yield, and ground tomato peels of 0.3 mm particle size and unground tomato peels of 1 mm particle size were used for SFE at 50 °C, 400 bar and a CO_2_ flow rate of 4 g/min for 105 min using a batch of 10 g. Their findings showed that the particle size of tomato peels affects the kinetics and yield of oleoresin extraction, and they concluded that the extract was enhanced by the smaller particle size of the dried tomato peels.

### 4.4. Influence of Extraction Time 

Extraction time is important for SFE because it can affect the extract’s composition. When the extraction time is short, it can lead to incomplete extraction. However, if the extraction time is too long, it would result in time and solvent wastage, as well as bioactive compound degradation [41]. The extraction time depends on flow rate: When the flow rate is high, the extraction time is short. A preliminary study should be performed to determine the optimal time and fluid flow rate to obtain the highest extract yield [50].

## 5. SFE in Plant Bioactive Compounds Extraction 

The use of natural substances in various industries such as food, pharmaceutical, and cosmetics has gained considerable attention recently due to their confirmed effects by the scientific researcher as sustainable alternatives to synthetic compounds, their perceived safety to the consumer, and because they are environmentally friendly. 

Plants symbolize a significant resource of chemical components that are historically used as natural remedies for some diseases and now those components are used in food additives production (e.g., natural flavor, aroma, and color), the creation of functional foods, production of natural pesticides, and development of new drugs [3]. Plant bioactive compounds are natural non-nutritive components of plant food biosynthesized as secondary metabolites [5]. They can provide essential benefits or undesirable effects on humans or animals and are found in small concentrations but are economically significant [57]. Plant bio-actives are usually identified in plant foods such as fruits, vegetables, grains, and non-food plants such as herbs, spices, aromatics, and, sometimes, in plant waste materials from factories. Even though they are not recognized as essential components, different experiments showed that they have a role in enhancing human health.

Different bioactive compounds extracted from natural sources by using supercritical CO_2_ extraction have been shown (Table 3) to exhibit antimicrobial, antioxidant, antiseptic [58,59], antibacterial, antifungal [60], antiviral, anti-inflammatory [2], antitumor [61], anti-obesity, anticholinesterase, phagocytotic, and therapeutic values [62,63]. They can also play the role of functional food constituents such as coloring, flavoring, preserving food additives, fragrances, authenticity indices, and biomarkers in metabolomic pathways. The synergism in some of the bioactive compounds enhances their bioactivity [64]. 

As the extraction process is the leading method to recover bioactive compounds, supercritical CO_2_ extraction has been reported as the best modern technology to sustainably and safely extract bioactive compounds.

**Figure 2:**
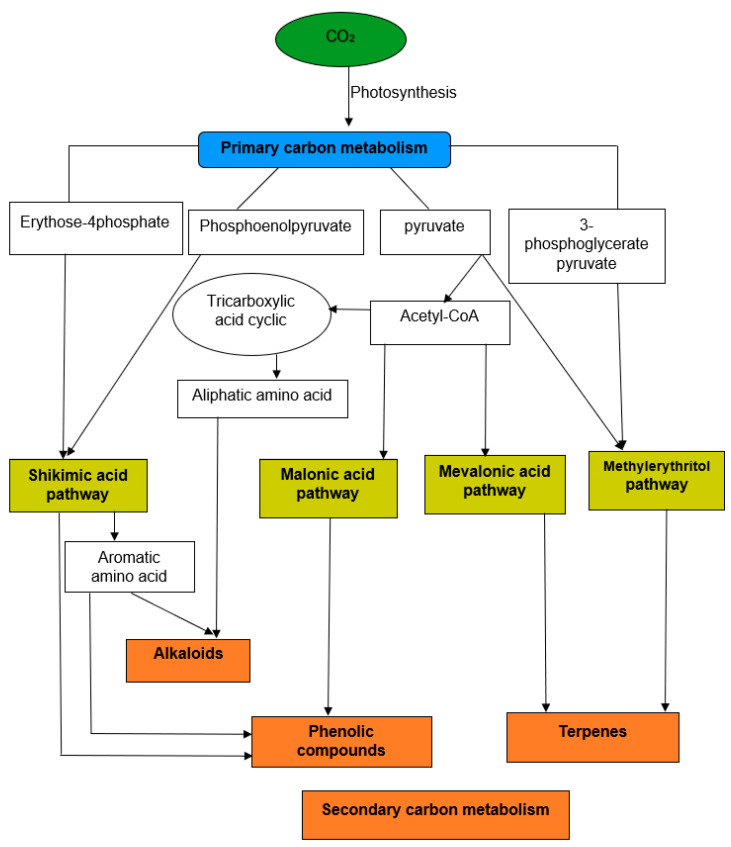
A general overview of the biosynthetic pathways involved in the biosynthesis of secondary metabolites (main categories of bioactive compounds) and their inter-relationships with primary metabolism in plants [3].

According to biochemical pathways and chemical classes, plant bioactive compounds are biosynthesized from four primary pathways: the shikimic acid pathway, malonic acid pathway, mevalonic acid pathway, and methylerythritol pathway [3,73]. From the pathways mentioned above, plants’ natural bioactive compounds can be categorized into three main classes: alkaloids, terpenoids/terpenes, and phenolics [5,73,74]. A simplified figure of diverse pathways for the biosynthesis of the three major groups of plant bioactive compounds is shown in Figure 2. 

Plant bio-actives are found in an enormous range of foods consumed as part of the human diet such as fruits, vegetables, chocolate, wine, tea, and coffee. However, those used in various industries can be extracted from different parts of plants such as leaves, stems, roots, tubers, seeds, buds, fruits, and flowers [2].

### 5.1. Alkaloids

Alkaloids are secondary plant metabolites, which are also found in microbes and animals. Structurally, they consist of carbon, hydrogen, nitrogen, and occasionally oxygen. Their alkaline nature is due to their nitrogen content [58]. There are over 12,000 known alkaloid compounds with low molecular weight, which have effects in the defense of plants against herbivores and pathogens. Some alkaloids exhibit significant bioactivities that has led to their exploitation as pharmaceuticals, stimulants, narcotics, and poisons [58,73]. Each plant species usually comprises a few alkaloids, but the most well-known alkaloids are morphine, codeine, caffeine, nicotine, cocaine, and vinblastine [73].

Caffeine is an excellent example of an alkaloid, commonly known to be present in different soft beverages, drinks, and some natural plants such as coffee beans, cocoa beans, mate leaves, guarana seeds, kola nuts, and tea leaves [75]. Several years ago, caffeine was employed as one of the ingredients in the production of some pharmaceuticals. However, various studies have indicated the side effects of caffeine consumption in humans. For example, insomnia, nervousness, irritability, anxiety, hostility, and mood swings, and sometimes it causes health problems in pregnant, children, and some patients [75]. These adverse consequences have resulted in the increased consumption of decaffeinated products that causes the evolution of various techniques that eliminate this alkaloid from coffee beans. Among the various techniques investigated, supercritical CO_2_ has been reported as an effective modern process to extract and eliminate caffeine from coffee, tea, and other products [63]. According to different research (Table 4), the extraction of alkaloids by using SC-CO_2_ extraction has been considered a quick and effective process compared to the conventional technique.

From the analysis of studies on alkaloid extraction with SC-CO_2_, the conditions of the parameters to be taken into consideration were different due to the plant material and working conditions. A pressure of 300 bar, temperature of 60 °C, and using ethanol as the co-solvent were considered the optimal parameters for that extraction.

### 5.2. Terpenes or Terpenoids

Terpenoids are among the most prominent groups of secondary metabolites, and over 25,000 members of this group have been reported [74]. The chemical structure of terpenoids is formed by head to tail rearrangement of two or more isoprene molecules. Isoprene is a basic structure of the terpenoids, made of a branched five-carbon unit synthesized from acetyl-CoA or 3-phosphoglycerate [80].

Repetitive combination of the five-carbon unit of isoprene generates numerous types of terpenoid molecules such as hemiterpene (C_5_), monoterpene (C_10_), sesquiterpene (C_15_), diterpene (C_20_), triterpene (C_30_), and tetraterpene (C_40_), etc. [80,81]. Terpenoids play essential physiological roles in plants including growth, development, and defense. Different terpenoids have been extracted using SC-CO_2_ (Table 5) and their applications are great and varied across industries.

In reference to studies on terpenoid extraction with SC-CO_2_, the parameters to be taken into consideration can be different due to the plant material and working conditions, but the temperature should be around 40 °C and the pressure should be 250 bar to obtain the highest yield, although the use of different co-solvents was not considered in some studies.

Since ancient times, terpenoids have been widely used by humans. Some monoterpenes and sesquiterpenes are used as food additives, and flavor and fragrance agent are added to food products, beverages, perfumes, soaps, toothpaste, tobacco and other product [81]. For example, carotenoids are a well-known class of tetraterpene plant pigments; they are responsible for the yellow-red colors of various plant organs, and they are used in the food industry as natural food colorants to make the food product color more appealing to consumers. Carotenoids are among the most important bioactive compounds which can enhance human health due to their pro-vitamin A activity, anti-cancer activities, and antioxidant power [92,99]. Carotenoids from various vegetables matrices, such as pumpkins, tomatoes, carrots, apricot, peach, sweet potato, green, yellow, and red peppers, have been successfully extracted by supercritical carbon dioxide [92]. In recent years, the demand for carotenoids has increased extensively because of the growth in its application as animal feed, dietary supplements, pharmaceuticals, food and beverages, cosmetics, etc. [92]. Carotenoid demand is expected to record an annual increase of 4% from 2018 to 2023 and exceed a value of the USD 2 billion by 2023 worldwide. The primary market is in Europe, representing 42% of the total, followed by North America and Asia, which represent 25% and 20% of the market, respectively [99,100]. 

Essential oils are among the most important compounds to have been extracted by supercritical CO_2_ from various species of herbs and aromatic plants, and they are generally composed of terpenes and terpenoids. They are used to produce food additives, cosmetics, herbicides, insecticides, medicines, fragrance in perfumery, and in aromatherapy [82]. Typically, essential oils can be found in every part of plants, however, their composition and effects may vary with the variety of the plants. Essential oils are always produced in small quantities, and their extraction yields can vary between 0.5–6%, but their positive effects do not require large quantities [101]. Essential oils have numerous beneficial properties including antifungal, anti-inflammatory, antibacterial, and antioxidant activity [102]. The antimicrobial effect of essential oils can be associated with their ability to penetrate the bacteria cell wall and inhibit its functional properties [84]. This ability is due to its hydrophobicity, which allows essential oils to separate the lipids from the cell membrane and cause an increase in cell permeability.

Currently, both academia and pharmaceutical industry are highly interested in essential oil extraction due to their pharmacological characteristics. The insecticidal activities of essential oils are also of interest to agricultural scientists and agri-businesses. Moderate pressures (90–120 bar) and temperatures (35–50 °C) are desirable to dissolve the essential oil compounds during extraction [102].

### 5.3. Phenolic Compounds

Phenolic compounds, also known as phenols, are secondary natural metabolites generally found in the plant kingdom (they are rarely present in bacteria, fungi, and algae) [103]. Approximately 8000 types of phenolics are known, for example, flavonoids, stilbenes, lignans, resveratrol, and gallic acid [74]. Biogenetically, phenolic compounds are metabolized either by the shikimic acid pathway where lignin is formed, or the malonic acid pathway, in which the major products are the simple phenol or both (shikimic and malonic), where most of flavonoids are formed [74,103]. They are symbolized by an aromatic ring with hydroxyl groups.

Phenolic compounds play an essential role in plants including growth, pigmentation, and reproduction protection against pests [103]. Several researchers have reported that consumption of phenolics can play a crucial role in human health by controlling metabolism, weight, chronic disease, and cell proliferation due to their antioxidant power and free radical scavenging properties, among other characteristics [73]. In food production, polyphenols can increase the shelf life of products by preventing oxidation due to their antioxidant activity, which relies mainly on the number and position of the hydroxyl groups related to the carboxyl functional group [73,103].

Flavonoids are one of the most well-known bioactives in the phenolics class. More than 4000 flavonoids have been discovered currently, and they are commonly present in fruits, seeds, leaves, bark, and flowers of plants. In plants, these compounds play a significant role in plant pigmentation, protecting them against pathogens [104,105]. Flavonoids are known for their antioxidant and chelating properties, which provide substantial health benefits such as the control and prevention of cancer, cardiovascular disease, and chronic inflammatory conditions [105]. To date, SFE (Table 6) has been established as an effective method to extract phenolic compounds from different plant materials.

From the studies listed in Table 6 regarding phenolic extraction with SC-CO_2_, the variables to be taken into consideration can differ due to several factors such as the plant material, targeted compounds, and working conditions. However, the optimal conditions were considered a pressure of 200 bar, temperature of 60 °C, and 5% ethanol as the co-solvent.

## 6. Current Applications of Supercritical Fluid Extraction 

Supercritical fluid-based technologies are involved in a broad spectrum of industrial applications that have experienced significant progress in recent years. Extraction with supercritical fluids has been applied extensively in the food, pharmaceuticals, and cosmetics industries.

### 6.1. Application of SC-CO_2_ in Food Industry

One of the primary trends in the food industry is the demand for all-natural food ingredients free of chemical additives. Natural food antioxidants are derivatives of plant by-products. Food waste valorization is under intense worldwide investigation topic and supercritical fluid extraction has been reported as one of the best ways to valorize agro-industry byproducts. Those extracts showed sustainable use in food industries as a good source of phenolic compounds with antioxidant activities [93,112,113,114,115]. For example, the extract of mango peel has been used as a natural antioxidant in sunflower oil to control lipid oxidation; the optimal conditions were 25 MPa, 60 °C and 15% ethanol in water by using a Box–Behnken design [92]. Supercritical fluid extraction is also applied in the extraction of cholesterol and other lipids from egg yolk [116], extraction of lipids and cholesterol from meat and meat products [117], decaffeination of coffee and tea [24,118,119], extraction of hops [71,120,121], extraction of bioactive compounds [65,91,101,102], extraction of free amino acids [122], extraction of lipids and cholesterol from fish [123], extraction of natural glycosides [124], fractionation of natural colorings, natural flavorings, and fragrances from several foodstuffs [99,125], and the separation of spices and essential oils [82,83,84,85,86].

### 6.2. Application of SC-CO_2_ in the Pharmaceuticals Industry

In the pharmaceuticals industry, development in the properties of active pharmaceutical ingredients is generally preferred. A significant challenge in this respect is particle size reduction, which increases bioavailability [126]. The primary use of supercritical fluids in the pharmaceuticals industry involves processes such as particle formation for drug delivery systems and crystal engineering; complexing cyclodextrins; coating, foaming and tissue engineering; impregnation [127] and purification of pharmaceutical excipients; and sterilization and solvent removal.

The best application of supercritical fluids in pharmaceuticals deals with the isolation of bioactive compounds from a mixture (purification from reactions, quantification of an active enantiomer, extraction from a natural matrix) [24]. Different plants have been used for the extraction of compounds to be used in pharmacy, such as *Catharanthus roseus*, a rich source of alkaloids, from which two dimeric alkaloids were extracted that are extensively used as antineoplastic drugs vinblastine and vincristine [79,126]. *Artemisia annua* L. exhibits a vigorous antimalarial activity due to the existence of artemisinin and its derivatives (like dehydroartemisinin). These compounds are particularly active against drug-resistant strains of *Plasmodium falciparum* [72]. *Melocactus zehntneri* is a medicinal cactus that is unusual pharmacologically because it contains isoquinoline and indole alkaloids, which are used in several drugs [128].

### 6.3. Application of SC-CO_2_ in the Cosmetics Industry

Cosmetics are daily use products that are currently available in a different market. Nowadays, people are interested in natural products, and they have realized that one of the most active ingredients of cosmetics is the antioxidant, which can interrupt radical-chain processes, help skin repair systems, improve cell rejuvenation and prevent skin-cancer [129]. Different research has shown that SC-CO_2_ extraction is a great technique that provides natural extracts with attractive fragrances or active ingredients that add value to the cosmetics by enhancing their practical action and attributes. SC-CO_2_ has been used for the isolation of antioxidants and parabens from cosmetic products [130]. Vogt et al. [131] investigated the extract of blackcurrant seeds, strawberry seeds, hop cones, and mint leaves obtained by SC-CO_2_ for the production of shower gels and shampoo, and the result showed that those natural extracts could be used as ingredients in shower gels.

## 7. Conclusions

The literature reviewed herein has shown that SFE is a separation technique commonly employed to extract natural bio-active compounds from plants due to the unique properties of supercritical fluids, that is, they simultaneously behave like a liquid and like a gas. The interest in supercritical fluid extraction is not only at the laboratory level as an analytical tool but also in industrial processing, mainly decaffeination of coffee or tea, extraction of essential oils, extraction of high added value compounds and fatty acids. Supercritical fluid extraction is favored due to its high selectivity, high efficiency, and short extraction time. Thus, in this review, factors influencing the supercritical fluid extraction process (temperature, pressure, and co-solvent) were discussed using examples. We assume that this technology will experience continued growth in the coming years and will be beneficial to sustainable development, given its green credentials, and will help to reduce the use of organic chemicals.

## Figures and Tables

**Table 1:** Chemical solvents employed in supercritical fluids extraction (SFE) and their critical characteristics [30].

Solvent	Molecular Weight	Critical Temperature	Critical Pressure	Critical Density
	[g/mol]	[K]	[MPa]	[g/cm^3^]
Carbon dioxide	44.01	304.1	7.38	0.469
Water	18.02	647.3	22.12	0.348
Methane	16.04	190.4	4.60	0.162
Ethane	30.07	305.3	4.87	0.203
Propane	44.09	369.8	4.25	0.217
Ethylene	28.05	282.4	5.04	0.215
Propylene	42.08	364.9	4.60	0.232
Methanol	32.04	512.6	8.09	0.272
Ethanol	46.07	513.9	6.14	0.276
Acetone	58.08	508.1	4.70	0.278

**Table 2:** Comparison of physicochemical properties of gases, supercritical fluids, and liquids [30].

State	Density [kg/m^3^]	Viscosity [µPa]	Diffusivity [mm^2^/s]
Gases P = 1 atm, T= 21 °C	1	10	1–10
Supercritical fluids P = Pc, T = Tc	100–1000	50–100	0.01–0.1
Liquids P = 1 atm, T = 15–30 °C	1000	500–1000	0.001

P = pressure, T = temperature.

**Table 3:** Selected bioactive compounds and their bioactivities.

Compounds	Plant Materials	Bioactivities	References
Geranylgeraniol	Annatto seed (*Bixa Orellana*)	anti-inflammatory activity, regulation of testosterone production, action against Chagas disease and leishmaniasis, and anti-cancer activity	[65]
Curcumin	Turmeric rhizomes (*Curcuma longa* L.)	antioxidant, antimalaria, antimicrobial, anti-viral properties, fungicidal activity, anti-Alzheimer anti-mutagenic, and anti-carcinogenic qualities	[66]
Thymol	Thyme (*Thymus praecox* *Polytrichus*)	antibacterial, antifungal, anti-inflammatory, antioxidant activities, local anesthetic	[67]
Eugenol	Purple basil (*Ocimum basilicum*)	antioxidant, antibacterial and antimicrobial activities	[68]
Carvacrol	*Satureja montana* L.	antioxidant, antiproliferative, and anti-cancer	[69]
Linalool	*Coriandrum sativum* L. and *Ocimum basilicum* L.	antimicrobial, anti-carcinogenic, antioxidant and antidiabetic activities	[69]
Camphor	Sage (Sage *officinalis*)	anti-inflammatory and anti-atherogenic	[70]
Xanthohumol	Hops (*Humulus lupulus*)	antibacterial activity anti-cancer	[71]
Artemisinin	*Artemisia annua* L.	antimalaria, antiulcerogenic, antifibrotic and antitumoral activity	[72]

**Table 4:** Application of supercritical CO_2_ for the separation of alkaloids from plants.

Compounds	Plant Material	SFE Parameters	Country	References
Pyrrolidine	Leaves of *piper amalago* (Piperaceae)	Pressure: 150, 200, and 250 bar Temperature: 40, 50, and 60 °C Co-solvents: ethanol, methanol and propyleneglycol 5% (*v*/*v*) Extraction time: 20, 40, and 60 min CO_2_ flow rate: 3 mL/min Particle size: 0.757 mm	Brazil	[76]
Olchicine, 3-demethylcolchicine, colchicoside	Seeds of wild plants of *Colchicum autumnale* L. (Colchicaceae)	Temperature: 25, 30, 35 and 40 °C Co-solvent: methanol 3% Extraction time: 0 to 25 min static extraction and 0 to 30 min dynamic extraction Density: 0.80, 0.85 and 0.90 g/mL CO_2_ flow rate: 1.5 mL/min	Spain	[59]
Total alkaloids, peimine, peiminine	Flower of Fritillaria thunbergii Miq	Pressure: 150–350 bar Temperature: 50–70 °C Co-solvent: ethanol and water ratio 80:100 (*v*/*v*) Extraction time: 90–210 min	China	[62]
Caffeine	Guayusa leaves (*Ilex guayusa* Loes)	Pressure: 150, 200 and 250 bar Temperature: 45, 60 and 75 °C Extraction time: 180 min CO_2_ mass flow: 8.3 g/min	Brazil	[60]
	Tea stalk and fiber waste	Pressure: 150–300 bar Temperature: 50–70 °C Co-solvent: ethanol 1–7 % Extraction time: 60–300 min CO_2_ flow rate: 10 g/min Particle size: 0.2–0.6 mm	Turkey	[77]
	Green coffee beans	Pressure: 152, 248 and 352 bar Temperature: 50 and 60 °C Co-solvent: ethanol and isopropyl alcohol 5% (*v*/*v*) CO_2_ flow rate: 1.8 g/min	Brazil	[78]
Vinblastine and vincristine	*Catharanthus roseus*	Pressure: 300 bar Temperature: 40, 50, and 60 °C Co-solvent: ethanol 2, 5 and 10% (*v*/*v*)	Brazil	[79]

**Table 5:** Examples of terpenoids extracted by supercritical CO_2_.

Targeted Extract	Plant Materials	SFE Parameters	Country	References
Essential oils	*Piper auritum*	Pressure: 103.4 and 172.4 bar Temperature: 40 and 50 °C	Mexico	[82]
	*Lippia graveolens*, (Mexican oregano leaves)	Pressure: 130, 150 and 350 bar Temperature: 40 and 60 °C	Mexico	[48]
Essential oils	Spearmint leaves	Pressure: 85–120 bar Temperature: 38–50 °C, CO_2_ flow rate: 0.059–0.354 g/min Particle size: 0.177–2 mm Dynamic extraction time: 20–120 min	Iran	[83]
	Roots of vetiver grass	Pressure: 145 bar Temperature: 45 °C Co-solvent: ethanol (0, 5, 10 and 15%)	Australia	[84]
	Clover leaf extract	Pressure: 150, 185 and 220 bar Temperature: 40, 50 and 60 °C	Brazil	[85]
	Rosemary (*Rosmarinus officinalis*)	Pressure: 103.4 and 172.4 bar Temperature: 40 and 50 °C Particle size: 0.6 mm	Mexico	[86]
	Ruta chalepensis	Pressure: 100, 150, 220 bar Temperature: 40 °C Extraction time: 30 min static extraction followed by 220 min of dynamic extraction	Tunisia	[87]
	*Echinophora platyloba*	Pressure: 80–240 bar Temperature: 35–55 °C Dynamic extraction time: 30–150 min Particle size: 0.30–0.90 mm	Iran	[88]
Raspberry seed oil	Raspberry seed	Pressure: 250, 300 and 350 bar Temperature: 40, 50 and 60 °C CO_2_ flow rate: 3, 5 and 6 g/min, Particle size: 0.2–0.4 mm Extraction time: 240 min	Serbia	[89]
Apple seed oil	Apple seed	Pressure: 300, 500, 750, 1000 and 1300 bar Temperatures: 43, 53 and 63 °C	New Zealand	[90]
Carotenoids	Nantes carrots peels	Pressure: 150, 250 and 350 bar Temperature: 50, 60 and 70 °C Co-solvent: ethanol 5, 10 and 15% (*v*/*v*) CO_2_ flow rate: 15 g/min Extraction time: 80 min	UK	[91]
	Flesh and peels of sweet potato, apricot, tomato, peach and pumpkin, and the flesh and wastes of green, yellow and red peppers	Pressure: 350 bar Temperature: 59 °C Co-solvent: ethanol 15.5% (*v*/*v*) CO_2_ flow rate: 15 g/min Extraction time: 30 min	UK	[92]
	Mango peels	Temperature: 40–60 °C Pressure: 250–350 bar Co-solvent: ethanol 5–15% (*v*/*v*) CO_2_ flow rate: 6.7 g/min Extraction time: 180 min	Colombia	[93]
Germacrene (sesquiterpene)	Leaves of *Piper klotzschianum*	Pressure: 180, 200, and 220 bar Temperature: 40, 60, 80 °C Co-solvent: methanol, ethanol, isopropanol (1, 3, 5%)	Brazil	[94]
Green coffee oil	Green coffee beans	Pressure: 200–400 bar Temperature: 40–60 °C Co-solvent: ethanol 0–5.7% (*v*/*v*)	Brazil	[95]
Vouacapan (diterpenes)	Sucupira fruits (*Ptedoron* spp.)	Pressure: 100–220 bar Temperature: 40–60 °C	Brazil	[96]
Oxygenated monoterpenes (camphor, 1,8-cineole), α-humulene, viridiflorol, and manool	*Salvia officinalis* L. (sage) leaves	Pressure: 100–300 bar Temperature: 40–60 °C Extraction time: 90 min	Croatia	[97]
Lycopene	Tomato peel by-product containing tomato seed	Temperature: 70–90 °C Pressure: 20–40 bar Particle size: 1.05 ± 0.10 mm CO_2_ flow: 2–4 mL/min Extraction time: 180 min	Japan	[98]
Geranylgeraniol	Annatto seed	Pressure: 100, 170, 240 and 310 bar Temperature: 40 and 60 °C CO_2_ densities: 290–915 kg/m^3^	Brazil	[65]
Artemisinin	*Artemisia annua* L.	Pressure: 100 bar Temperature: 40 °C CO_2_ flow rate: 13.3–20 g/min	Italy	[72]

**Table 6:** Examples of phenolics extracted by supercritical CO_2._

Compounds	Plant Material	Studied Parameters	Country	References
Flavonoids	*Strobilanthes crispus* (Pecah Kaca) leaves	Pressure: 100, 150 and 200 bar Temperature: 40, 50 and 60 °C Dynamic extraction time: 0, 40, 60 and 80 min	Malaysia	[104]
	*Odontonema strictum* leaves	Pressure: 200 and 250 bar Temperature: 55–65 °C CO_2_ flow: 15 g/min Co-solvent: ethanol 95% Extraction time: 210–270 min	Burkina Faso	[106]
Flavonoids (*Tiliroside*)	*Tilia* L. flower	Pressure: 100–220 bar Temperature: 45–80 °C Time: 20–60 min	Poland	[107]
Total phenolic compounds, total flavonoids	Radish leaves	Pressure: 300 and 400 bar Temperature: 35, 40, and 50 °C CO_2_ flow rate: 10 g/min Co-solvent: ethanol	Argentina	[34]
Flavonoids (hesperidin, nobiletin, and tangeretin)	Citrus genkou peels	Pressure: 100–300 bar Temperature: 40–80 °C	Japan	[108]
Total phenols	Strawberry (*Arbutus unedo* L.)	Pressure: 150, 250, 350 bar Temperature: 40, 60, 80 °C Co-solvent: ethanol (0, 10, 20%) CO_2_ flow rate: 15 g/min Extraction time: 60 min	Turkey	[46]
Fatty acid	Pomegranate seed oil	Pressure: 240, 280 and 320 bar Temperature: 40,50 and 60 °C CO_2_ flow rate: 133.3 g/min Extraction time: 180 min	Italy	[42]
	Yacon leaves	Pressure: 150–250 bar Temperature: 30–70 °C Co-solvent: ethanol	Brazil	[109]
Phenolic compounds	*Hibiscus sabdariffa*	Pressure: 150–350 bar Temperature: 40 to 60 °C Co-solvent: ethanol 7–15% Total flow: 25 g/min Extraction time: 90 min	Spain	[110]
Tocotrienols	Annatto seed	Pressure: 100, 170, 240 and 310 bar Temperature: 40 and 60 °C CO_2_ densities: 290–915 kg/m^3^	Brazil	[65]
Tocopherol	Quinoa (*Chenopodium quinoa* Will)	Pressure: 200–400 bar Temperature: 40–60 °C	Spain	[111]
